# Functional role and epithelial to mesenchymal transition of the miR-590-3p/MDM2 axis in hepatocellular carcinoma

**DOI:** 10.1186/s12885-023-10861-y

**Published:** 2023-05-04

**Authors:** Alaa Ibrahem Youssef, Gehad Mahmoud Khaled, Asma Amleh

**Affiliations:** 1 Department of Biotechnology, School of Sciences and Engineering, The American University in Cairo, New Cairo, 11835 Egypt; 2 Department of Biology, School of Sciences and Engineering, The American University in Cairo, New Cairo, 11835 Egypt

**Keywords:** miR-590-3p, MDM2 knockdown, miR-590-3p mimics, Transwell migration, Colony formation

## Abstract

**Background:**

There is considerable evidence that microRNAs (miRNAs) regulate several key tumor-associated genes/pathways and may themselves have a dual regulatory function either as tumor suppressors or oncogenic miRNA, depending on the tumor type. MicroRNA-590-3p (miR-590-3p) is a small non-coding RNA involved in the initiation and progression of numerous tumors. However, its expression pattern and biological role in hepatocellular carcinoma (HCC) are controversial.

**Results:**

In the current work, computational and RT-qPCR analysis revealed that HCC tissues and cell lines exhibited miR-590-3p downregulation. Forced expression of miR-590-3p attenuated HepG2 cells proliferation, migration, and repressed EMT-related gene expression. Bioinformatic, RT-qPCR, and luciferase assays revealed that MDM2 is a direct functional target of miR-590-3p. Moreover, the knockdown of MDM2 mimicked the inhibitory effect of miR-590-3p in HepG2 cells.

**Conclusion:**

We have identified not only novel targets for miR-590-3p in HCC, but also novel target genes for miR590-3p/MDM2 pathway in HCC like *SNAIL*, *SLUG*, *ZEB1*, *ZEB2*, and *N-cadherin*. Furthermore, these findings demonstrate a crucial role for MDM2 in the regulatory mechanism of EMT in HCC.

**Supplementary Information:**

The online version contains supplementary material available at 10.1186/s12885-023-10861-y.

## Background

Hepatocellular carcinoma (HCC) is the primary malignancy of liver cancer, and accounts for 75%-85% of all reported cases of liver cancer reported cases [1, 2]. The major causes of fatality in HCC patients are recurrence and metastasis, with 68% of patients with HCC develop this metastatic disease [3–5]. Hepatocellular carcinoma (HCC) is a major health issue in Egypt. The Egyptian National Cancer Registry (NCR) reports that HCC is the most frequently observed cancer in lower and middle Egypt, and the second most frequent in upper Egypt [6]. This difference in prevalence may be due to the higher prevalence of hepatitis C viral infection (HCV) in the Nile delta region (lower Egypt), which decreases as we move south [7]. In 2018, Egypt had one of the highest age-standardized mortality rates for HCC worldwide, along with Mongolia [8]. Therefore, elucidating the underlying mechanisms of HCC development and progression and identifying new biomarkers may help reduce the disease burden by enabling early diagnosis and highlighting avenues for the development of therapeutics, finally improving the prognosis of patients with liver cancer.

MicroRNAs comprise a group of small non-coding RNAs 18–22 nucleotides in length, which play a vital role in the regulation of gene expression [9]. There is growing evidence indicates that miRNA dysregulation can serve as a biomarker for the early diagnosis of HCC [10, 11]. miR-590-3p is a human intragenic miRNA located within the introns of the eukaryotic initiation factor 4H (*EIF4H*) gene. In the context of cancer, dysregulation of the miR-590-3p signature has been shown to play a dual regulatory role by enabling this miRNA to act as an oncomiR or tumor-suppressor depending on the specific tissue it is expressed in [12–14]. However, the expression and mechanistic role of miR-590-3p in HCC remain elusive, with contradictory reports [15–20] and warranting further study.

The proto-oncoprotein murine double minute 2 (MDM2) is overexpressed in several tumors, including HCC, and has been shown to have roles both dependent and independent of p53 [21, 22]. Recent reports have indicated that MDM2 expression in HCC is correlated with increased malignancy, epithelial-mesenchymal transition (EMT) progression, higher degree of invasiveness, and greater metastatic potential [23–25]. However, the key roles of MDM2 and detailed mechanisms in HCC are still far from clear and to the best of our knowledge, the regulatory function of miR-590-3p towards MDM2 expression has not been investigated.

The present study investigates the role of mir-590-3p in HepG2 cells; specifically, whether miR-590-3p regulates the proliferation, migration, and EMT progression of HepG2 cells. To further explore the underlying mechanism, we selected MDM2 for further study because Insilco analysis and luciferase assay suggested it to be a direct functional target of miR-590-3p. In addition, we knocked down MDM2 expression to characterize its role in HepG2 cells. Together, the findings of these assays illustrate the role of the miR-590-3p/MDM2 axis in HCC.

## Materials and methods

### In silico analysis

The differential expression data of miR-590-3p and MDM2 between HCC and normal liver tissues were extracted from The Cancer Genome Atlas (TCGA) through Encyclopedia of RNA Interactomes (ENCORI) Pan-cancer analysis platform [26]. Potential downstream targets of miR-590-3p were predicted using the Condition-Specific miRNA Targets (CSmiRTar) database [27], which collects data from four common prediction tools (such as miRDB, TargetScan, microRNA.org and DIANA-microT) and then applies a functional filter to detect targets that are only expressed in a certain tissue and/ or a certain disease. TargetScan platform was used to feature the sequence alignment between miR-590-3p seed region and the downstream targets [28].

### Cell culture

Two adherent hepatocellular carcinoma cell lines were used in this study: HepG2 and SNU449. HepG2 is a well differentiated, non-invasive HCC cell line. SNU449 cell line is a tumorigenic and more advanced stage of HCC with grade (II–III) [29]. HepG2 was obtained from NAWAH Scientific Inc., (Cairo, Egypt) and SNU449 was a kind gift from Dr Mehmet Ozturk from the Department of Molecular Biology and Genetics, Bilkent University, Turkey. HepG2 and SNU449 were grown in DMEM and RPMI 1640, respectively (LONZA, Bend, OR, USA), supplied with 10% fetal bovine serum (GIBCO, Grand Island, NY, USA) and 5% penicillin–streptomycin antibiotic (GIBCO, Grand Island, NY, USA). Cells were maintained in a humidified incubator supplied with 5% CO2 at 37 °Ϲ.

### RNA Interference

The knockdown of the *MDM2* gene (NCBI Reference Sequence: NG_016708.1) was achieved using ON-TARGETplus SMARTpool siRNA (siMDM2 SMART Pool; L-003279–00-0020) (Dharmacon, Lafayette, CO, USA). Target sequences of the MDM2 siRNAs are presented in Table 1. AllStars Negative Control siRNA (SI027280) (Qiagen, Hilden, Germany), hidden sequence.

**Table 1:** ON-TARGETplus SMARTpool siRNA sequences (5’-3’)

siRNA 1	GCCAGUAUAUUAUGACUAA
siRNA 2	GAACAAGAGACCCUGGUUA
siRNA 3	GAAUUUAGACAACCUGAAA
siRNA 4	GAUGAGAAGCAACAACAUA

### Cell transfection

HepG2 cells were ectopically transfected with 40 nM of miR-590-3p mimics (Invitrogen, Waltham, MA, USA) or Allstar negative control siRNA (Qiagen, Hilden, Germany) using lipofectamine 3000 (Invitrogen, Waltham, MA, USA) in a 6-well plate with a seeding density of 4 × 10^5^. For MDM2 knockdown, cells were transfected with 60 nM of MDM2 siRNA or Allstar negative control siRNA in a 12-well plate with a seeding density of 7 × 10^4^. Cells were incubated for forty-eight hours before performing further experiments. All transfection experiments were done in an RNase-free environment.

### Real Time Quantitative Polymerase Chain Reaction (RT-qPCR)

To assess miR-590-3p expression levels, total RNA-enriched small RNAs were extracted using miRNeasy mini kit (Qiagen, Hilden, Germany). MiRCURY LNA SYBR Green qPCR System (Qiagen, Hilden, Germany) was carried out to assess the levels of miR-590-3p in HepG2 and SNU449 cells. In brief, 10 ng of mature miRNA was polyadenylated and reverse-transcribed parallelly into a universal one first-strand cDNA. A UniSp6 RNA spike-in was used in the RT reaction to control efficient cDNA synthesis. RT-qPCR was performed using Locked Nucleic Acid (LNA) SYBR Green qPCR assays. Mir-103a-3p served as the endogenous normalizing control.

For assessing mRNA levels of downstream genes used in this study, total RNA was extracted from transfected cells using Trizol reagent (Invitrogen, Waltham, MA, USA) as per the manufacturer’s protocol. RT reactions were made using 2 μg of total RNA samples using RevertAid First Strand cDNA synthesis kit (Thermo Scientific, Waltham, MA, USA) according to the manufacturer’s instructions. 5 ng/reaction of cDNA along with 250–300 nM forward or reverse primers were used to carry out the qPCR using PowerUp SYBR Green kit (Applied Biosystems, Waltham, MA, USA). GAPDH was used for normalizing the expression of different genes. Table 2 lists the primers used in this study. All qPCR was done on Applied Biosystems 7500 system.

**Table 2:** RT-qPCR primers used in the RT-qPCR analysis

Gene	Primer sequence
hsa-mir-590-3p	5'UAAUUUUAUGUAUAAGCUAGU
hsa-mir-103a-3p	5'AGCAGCAUUGUACAGGGCUAUGA
*GAPDH*	F: 5’-AAGGTCATCCCTGAGCTGAAC- 3’ R: 5’-ACGCCTGCTTCACCACCTTCT-3’
*MDM2*	F: 5’-CCCAAGACAAAGAAGAGAGTGTGG-3’ R: 5’-CTGGGCAGGGCTTATTCCTTTTCT-3’
*N-Cadherin*	F: 5’-GCGTCTGTAGAGGCTTCTGGT-3’ R: 5’-TCTGCAGGCTCACTGCTCTC-3’
*Vimentin*	F: 5’-GAACGCCAGATGCGTGAAATG-3’ R: 5’-CCAGAGGGAGTGAATCCAGATTA-3’
*SNAIL*	F: ACTATGCCGCGCTCTTTCCT R: GCTGCTGGAAGGTAAACTCTGG
*SLUG*	F: CAAGGCGTTTTCCAGACCCTG R: AAGAAAAAGGCTTCTCCCCCGT
*ZEB1*	F: TGCTGGGAGGATGACACAGG R: CTGCTTCATCTGCCTGAGCTT
*ZEB2*	F: TTCCTGGGCTACGACCATACC R: CAAGCAATTCTCCCTGAAATCC

### Transwell migration assay

To test the effect of mir-590-3p overexpression on the migration of HepG2 cells, Transwell assay was performed. Cells were collected forty-eight hours post-transfection, and almost (5 × 10^5^) of mimic or negative control transfected cells were suspended in media supplemented with 1% FBS. For MDM2 knockdown, almost 150*10^3^ cells/well of MDM2 siRNA or negative control.transfected cells were resuspended in serum-free media. Cell suspensions were loaded on the upper chamber of 8 μm cell culture translucent inserts (Greiner Bio-One, Frickenhausen, Germany) placed in a 24-well plate. Inside the 24-well plate, complete media with 10% FBS was added to serve as a chemotactic agent to allow for cell migration through the microporous filter. After 10 h, cells in the upper chamber of the insert were removed with cotton swabs. Migrated cells at the bottom of the insert were fixed with 4% formaldehyde and stained with DAPI (KPL, Gaithersburg، MD, USA) (1:1000 in PBS). Cells were examined under a fluorescent microscope at 20X magnification, and five random fields per insert were captured. Cells were counted using Fiji Image J software.

### Colony formation assay

A colony-formation assay was conducted to test if mir-590-3p has a role in regulating HCC clonogenicity. Forty-eight hours post-transfection, HepG2 cells transfected with either miR-590-3p mimic or negative control were detached and seeded in a 6-well plate at a density of 500 cells. Cells were incubated to colonize for seven days. For MDM2 knockdown, transfected cells were incubated for ten days. When visible colonies were seen under the microscope, the experiment was terminated, and formed colonies (more than 50 cells/ colony) were fixed with 4% formaldehyde, stained with crystal violet, and manually counted.

### Plasmid construction and luciferase reporter assay

Wild type (WT) and mutant (MUT) constructs of the 3′ untranslated region (3′UTR) of MDM2 were chemically synthesized. For the WT constructs, the miR-590-3p binding site in the 3′UTR of MDM2 was cloned into the pmirGLO dual luciferase miRNA target expression vector (Promega, Madison, WI, USA). For the MUT constructs, the binding region nucleotides in the 3′UTR of MDM2 was completely deleted. The forward and reverse primer sequences of both designed constructs flanked by *SalI* and *NheI* restriction sites are shown in Table 3. HepG2 cells were co-transfected with 50 ng of the WT or MUT constructs along with miR-590-3p mimics or negative control using Lipofectamine 3000 (Invitrogen, Waltham, MA, USA). Twenty-four hours after transfection, cell lysates were prepared, and the relative luciferase activity was normalized to the total protein content and tested through a dual-luciferase reporter assay system (Promega, Madison, WI, USA).

**Table 3:** The forward (F) and reverse (R) primer sequences for WT and MUT 3′UTR constructs of MDM2

MDM2 3’UTR WT	F 5’ **CTAGC**GGCTAGTGATATATATAAAGTAAAATTTTCTTTGCAG**G** 3’
	R3’ **G**CCGATCACTATATATATTTCATTTTAAAAGAAACGTC**CAGCT** 5’
MDM2 3’UTR MUT	F5’ **CTAGC**GGCTAGTGATATATATAAAG–––-TTCTTTGCAG**G** 3’
	R3’ **G**CCGATCACTATATATATTTC–––-AAGAAACGTC**CAGCT** 5’

### Western blotting

Transfected cells were resuspended and lysed using the CelLytic™ M Cell lysis Reagent (Sigma Aldrish, Burlington, MA, USA). Equal amounts (15 μg) of proteins were loaded on 10% SDS–Polyacrylamide gel and separated by electrophoresis at 150 V for an hour. SDS gels were blotted to nitrocellulose membranes (GE Healthcare, Chicago, Illinois, USA). Membranes were then left for overnight blocking at 4 C in 5% nonfat dry milk diluted in 1X Tris-buffered saline with 0.01% Tween 20 (TBST). Following blocking, membranes were incubated with primary antibodies. Membranes were then washed by TBST and incubated with alkaline phosphatase-conjugated secondary goat anti-rabbit IgG or goat anti-mouse IgG antibodies (KPL, Gaithersburg، MD, USA) (1:10,000 in 5% non-fat milk). Finally, membranes were washed and incubated with the chromogenic substrate BCIP/NBT (KPL, Gaithersburg، MD, USA) until protein bands were visible with eye. Primary antibodies mentioned here are Anti-GAPDH (MA5-15,738, 1:10,000) (Invitrogen, Waltham, MA, USA) and MDM2 (E-AB-31995, 1:200) (Elabscience Biotechnology Inc., Houston, Texas, USA).

### Data analysis

The relative expression of miR-590-3p in HepG2 and SNU449 and the differential expression levels of downstream genes in different transfection conditions were analyzed using the comparative ΔΔCT method [30]. Fiji ImageJ software [31] was used to analyze Transwell assay results. Data presented as the mean ± standard error of the mean (SEM) from three independent experiments unless specified otherwise. For statistical analysis, an unpaired Students’ *t-*test was used to measure differences between two experimental groups. A probability value (*P*-value) < 0.05 was considered a significant result. Statistical analyses were established using GraphPad Prism 6 statistical packages [32].

## Results

### miR-590-3p is a downregulated miRNA in hepatocellular carcinoma

**Fig. 1:**
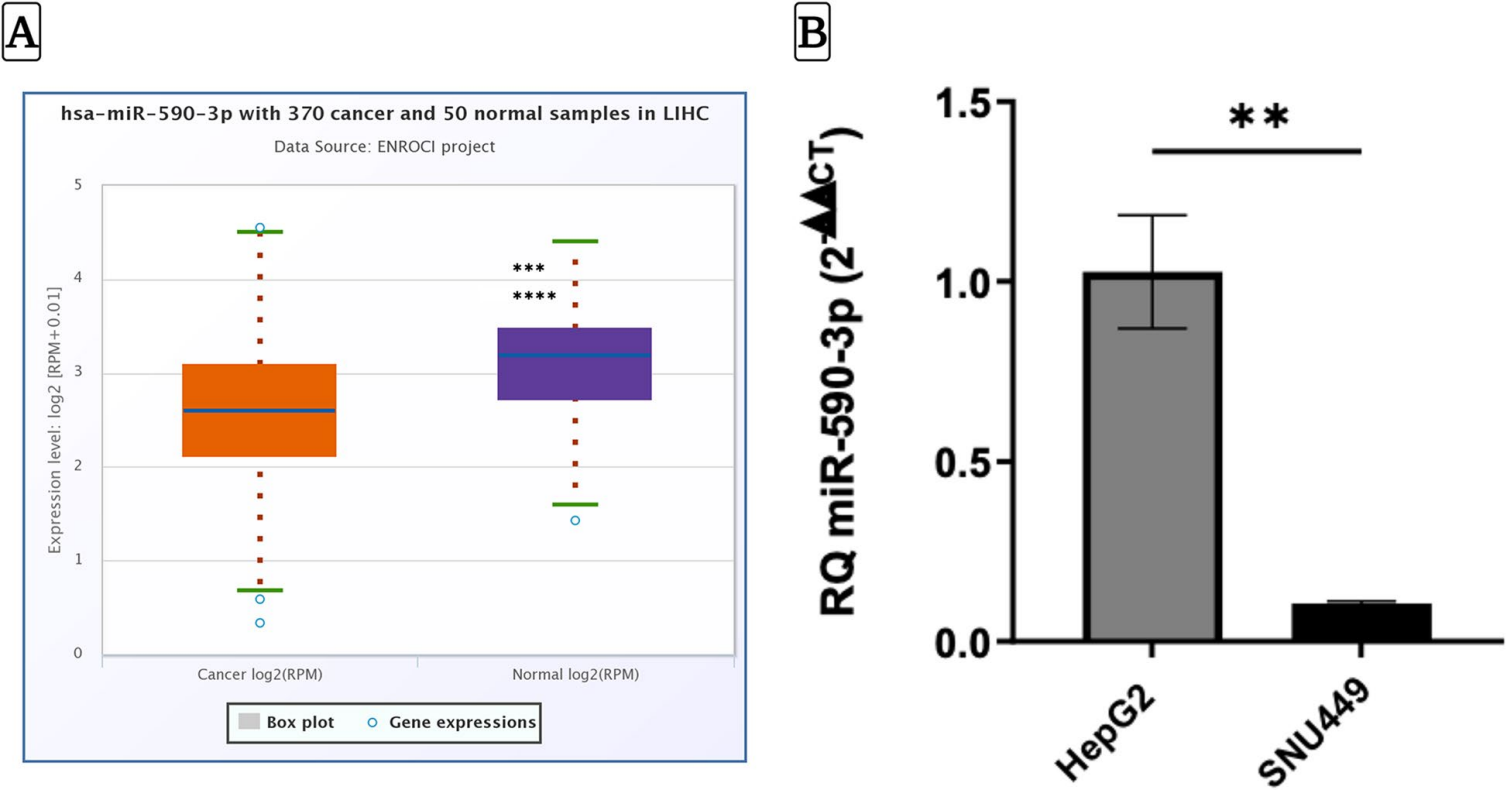
The downregulation pattern of hsa-miR-590-3p in hepatocellular carcinoma.** A** miR-590-3p differential expression between HCC (*n* = 370) relative to the normal liver tissues (*n* = 50). **B** MiR-590-3p is significantly lower in the SNU449 cell line compared to HepG2 cells, as shown by RT qPCR results. Data represents the mean ± SEM (*N* = 3). *P*-value was depicted using Student’s t-test. (** *P* < 0.01, **** *P* < 0.0001)

Comparison of miR-590-3p expression in HCC and non-cancerous liver tissues revealed a very significant (P > 0.0001) downregulation of miR-5909-3p expression in 370 HCC samples compared with 50 healthy counterparts, with a fold change of (0.75) (Fig. 1A). Examination of miR-590-3p levels in two cell lines representing an early and an advanced stage of HCC using RT qPCR revealed the expression of miR-590-3p to be significantly lower in SNU449 cells compared with HepG2 cells (*P* < 0.01) (Fig. 1B).

### Restoration of miR-590-3p expression inhibits cellular clonogenicity, migration, and epithelial-mesenchymal transition in HepG2 cells

**Fig. 2:**
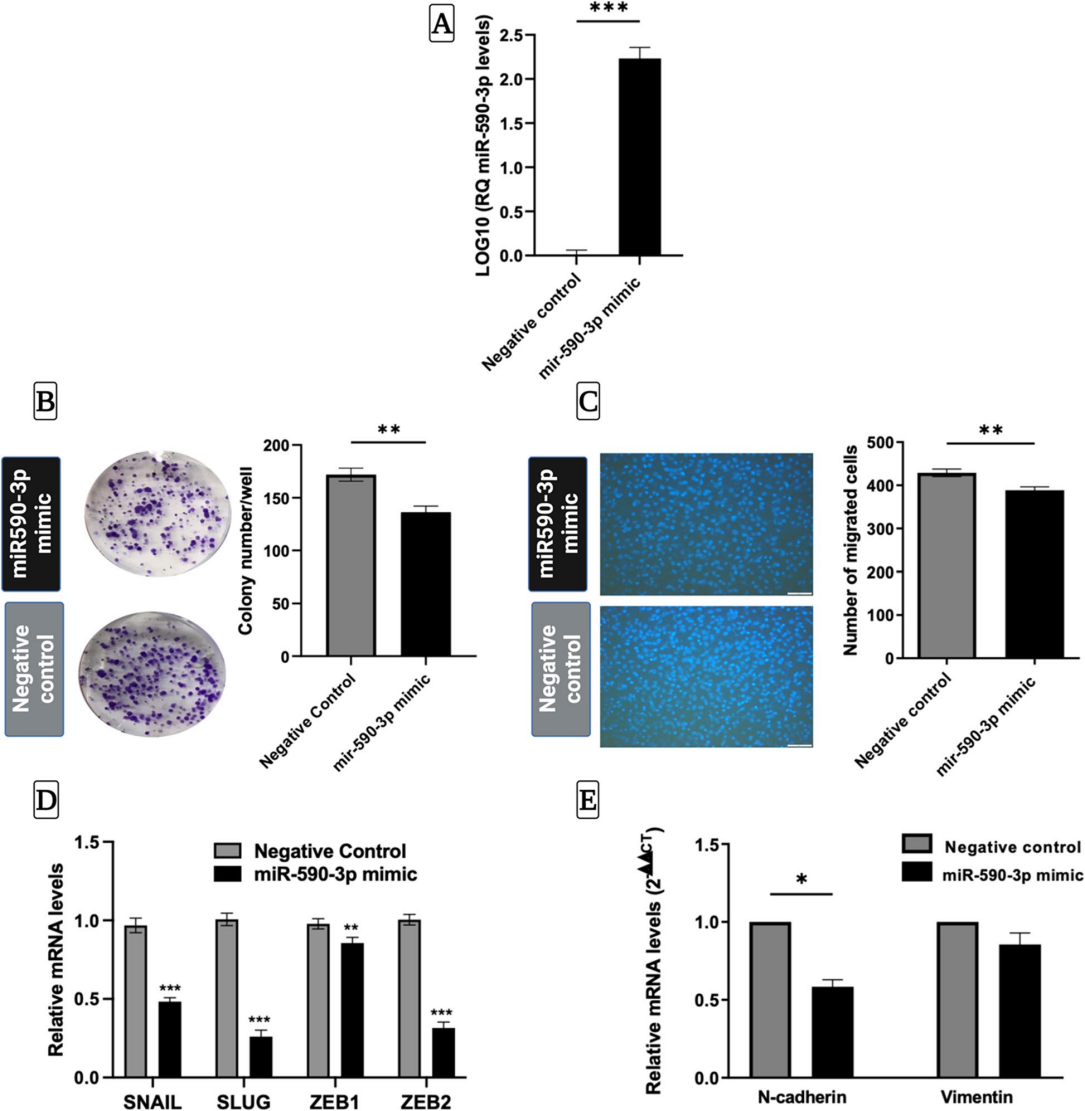
miR-590-3p suppresses cellular clonogenicity, migration and EMT in HepG2 cells. **A** The transfection efficiency of miR-590-3p mimics compared to negative control **B** Overexpressing miR-590-3p significantly decreased the number of HepG2 colonies compared to the negative control. **C** Overexpressing miR-590–3 showed a low but significant reduction in the number of migrated cells as compared to the negative control in the HepG2 cell line. **D** overexpressing miR-590-3p caused a significant decrease in the mRNA levels of *SNAIL, SLUG, ZEB1, and ZEB2.* **E** *N-cadherin* levels were significantly lower in miR-590-3p mimics transfected cells, while *Vimentin* levels were not significantly changed. Images were taken at 20X magnification using a fluorescent microscope and counted using Fiji Image J software. Data represents the mean ± SEM. (*N* = 2 for qPCR, *N* = 3 for Transwell and colony assay). *P*-value was depicted using Student’s t test. (* *P* < 0.05, ** *P* < 0.01, *** *P* < 0.001)

After establishing a miR-590-3p transient transfection using miR-590-3p mimics in the HepG2 cell line, miR-590-3p levels post-transfection were dramatically higher in miR-590-3p mimic transfected cells than in the negative control (Fig. 2A). Overexpression of miR-590-3p caused a significant decrease in the number of colonies of HepG2 cells compared with the negative control (Fig. 2B). The Transwell migration assay (Fig. 2C) revealed the cellular migration capacity of HepG2 cells to be significantly reduced when miR-590-3p was overexpressed. Having observed that miR-590-3p overexpression exerted a critical role in suppressing HepG2 cell motility, we investigated its effect on EMT. Bioinformatic analyses and RT qPCR revealed that the transcript levels of four EMT transcription factors: SNAIL, SLUG, ZEB1, and ZEB2 were dramatically decreased in miR-590-3p-mimics-transfected HepG2 cells compared with the control group (Figures S1 and 2D). In addition, a significant reduction was found in the levels of the mesenchymal marker N-cadherin, but not Vimentin, in HepG2 cells after overexpressing miR-590-3p (Fig. 2E). Consistently with qPCR results, a complementary binding between the seed region of miR-590-3p and *N-cadherin* was shown, but no sequence alignment was found with *Vimentin* (Fig. S2).

### *MDM2* is a specific downstream target gene of miR-590-3p in hepatocellular carcinoma

The CSmiRTar prediction algorithm identified five validated target genes that are liver-tissue and HCC-disease specific (Table 4). Among these, we selected MDM2 for further investigation based on a previous study that reported a functional network between MDM2 and miR-590-3p in HepG2 cells [33]. Specifically, miR-590-3p was identified as one of 33 P53-regulated miRNAs after doxorubicin treatment, and *MDM2* was shown to be a direct target gene of miR-590-3p. However, our study was primarily interested in exploring the P53-independent effects of MDM2, particularly those related to EMT.

**Table 4:** Alignment features of five experimentally validated potential targets of miR-590-3p in hepatocellular carcinoma predicted by the CSmiRTar algorithm

Gene Name	Average Normalized Score^a^	Validation method	References
*SETD7*	0.555	PAR-CLIP^b^	[34]
*RACGAP1*	0.444	PAR-CLIP^b^	[34]
*YWAHZ*	0.399	PAR-CLIP^b^	[35]
*RAPGEF1*	0.383	PAR-CLIP^b^	[36]
*MDM2*	0.239	PAR-CLIP^b^	[37]

^a^Average of scores from four prediction databases (higher scores indicate better predictions)

^b^PAR-CLIP refers to Photoactivatable Ribonucleoside-enhanced Cross-linking Immunoprecipitation, a technique used to identify RNA targets of RNA-binding proteins

**Fig. 3:**
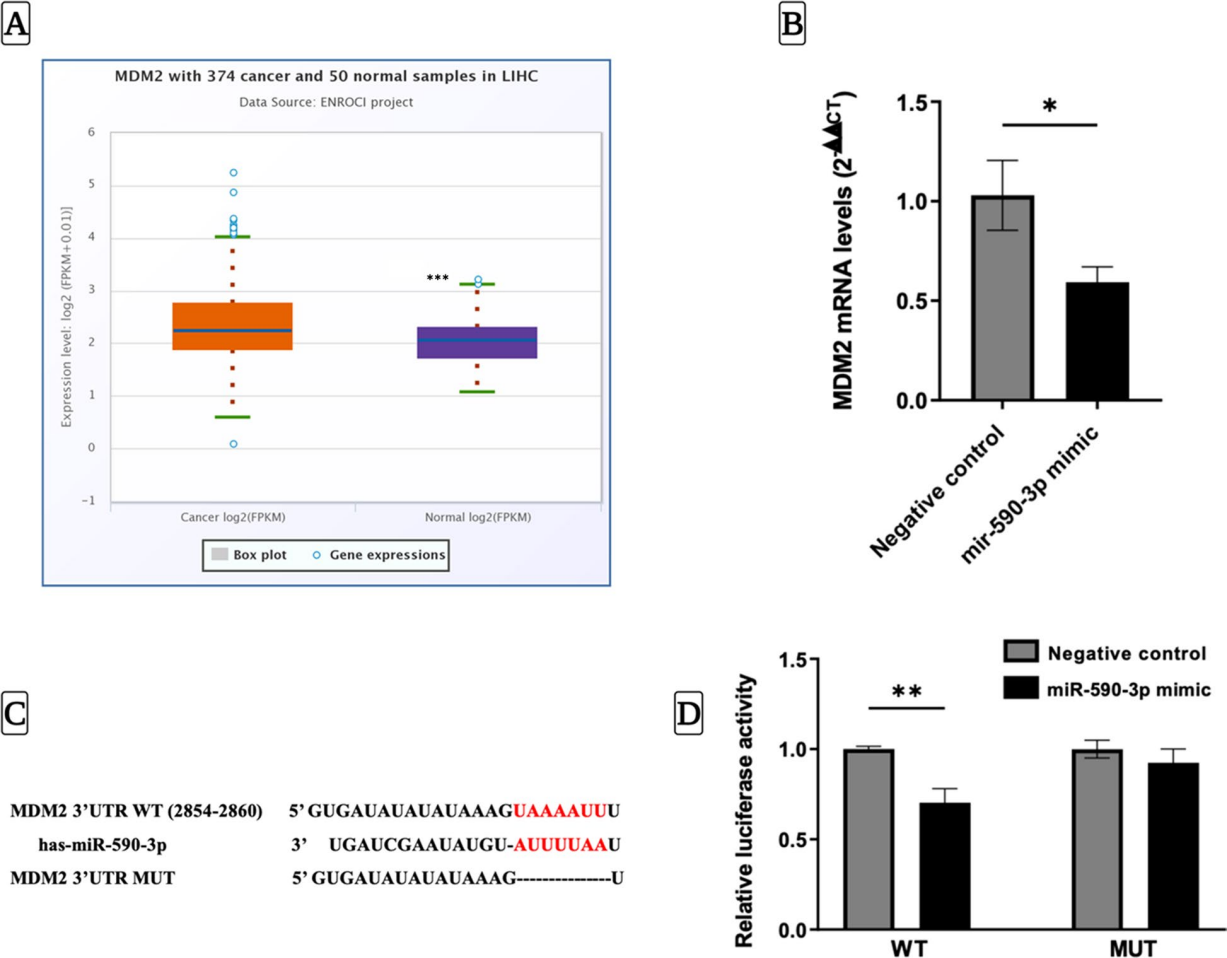
*MDM2* is a novel direct target gene of miR-590-3p in HCC.** A** Differential expression of MDM2 between normal and HCC tissues as obtained from TCGA data using Pan-cancer analysis platform. **B** The mRNA levels of *MDM2* in HepG2 cells transfected with miR-590-3p in mimic compared to the negative control. **C** The sequence alignment between MDM2 and miR-590-3p. **D** Relative luciferase activity of miR-590-3p mimic or negative control on the WT or MUT versions of MDM2. Data represents the mean ± SEM. (*N* = 3 for qPCR, *N* = 2 for luciferase assay). *P*-value was depicted using Student’s t-test. (* *P* < 0.05, ** *P* < 0.01, *** *P* < 0.001)

To further investigate the potential role of MDM2 in HCC, we analyzed TCGA data and found that MDM2 expression was significantly upregulated in HCC samples compared to healthy samples (fold change = 1.34, *P* < 0.001) (Fig. 3A). Additionally, we conducted functional experiments in HepG2 cells and found that overexpression of miR-590-3p significantly downregulated *MDM2* mRNA levels (*P* < 0.05) (Fig. 3B). We confirmed the target site of miR-590-3p on *MDM2* mRNA using TargetScan, which identified a sequence alignment in the seed region of miR-590-3p (Fig. 3C). Finally, to directly test if there is a mechanistic interaction between miR-590-3p and MDM2, we performed luciferase reporter assays and found that co-transfection of miR-590-3p mimics and WT constructs of the MDM2 3′UTR significantly decreased luciferase activity compared to the negative control (*P* < 0.01), while no change was observed in the MDM2 3′UTR MUT group (Fig. 3D). Taken together, our results provide evidence that miR-590-3p directly targets MDM2 and regulates its expression in HCC cells.

### Knockdown of MDM2 has similar effects as miR-590-3p overexpression in HepG2 cells

**Fig. 4:**
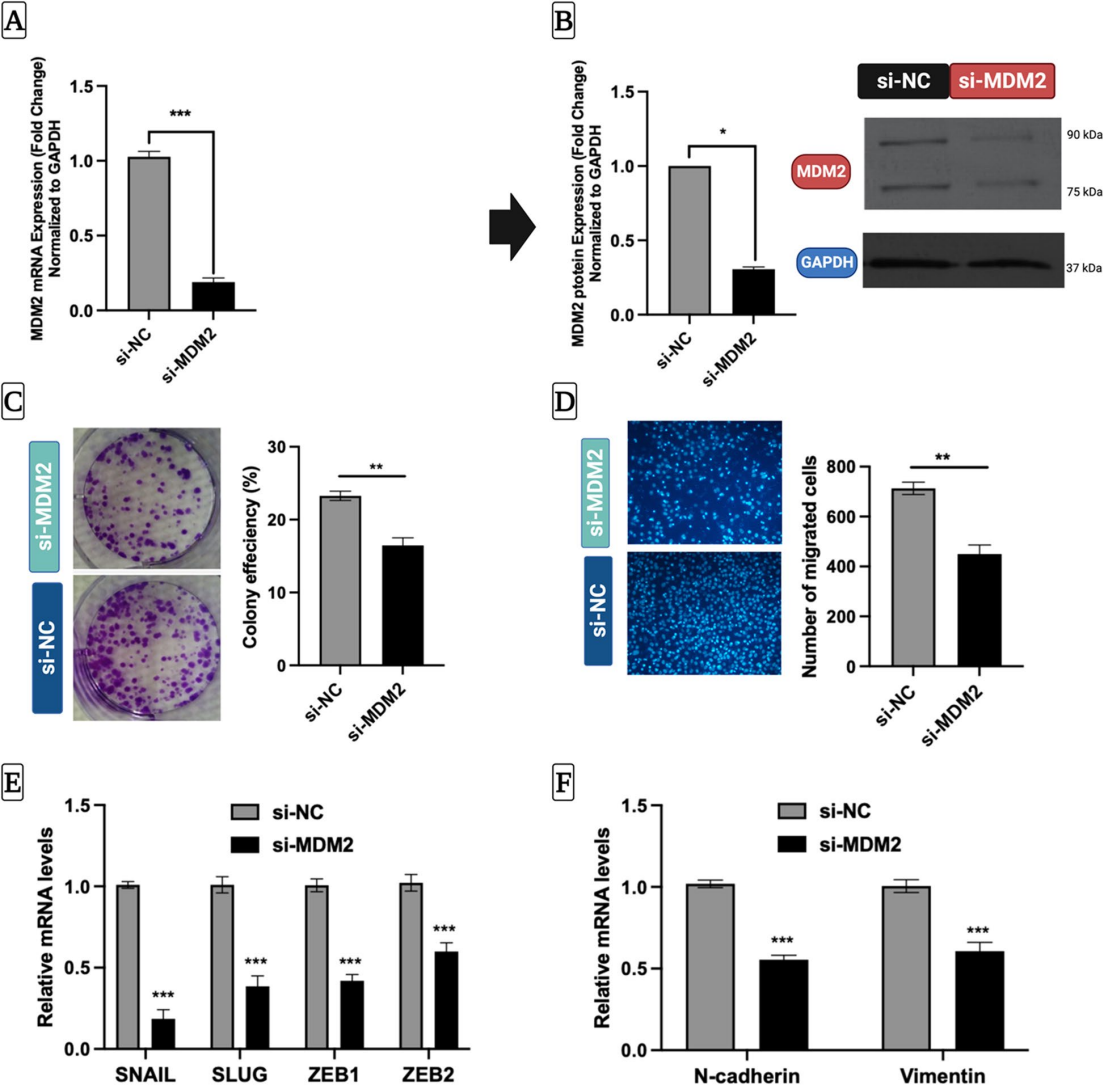
Inhibition of MDM2 mimics the tumor suppressor effect of miR-590-3p.** A** RT-qPCR showing the expression of *MDM2* after transfecting HepG2 cells with si-MDM2 or si-NC. **B** Western blot showing the successful depletion of MDM2 protein expression. Full-length blot is presented in Supplementary Figure S3(A&B). **C** Colony formation assay was performed to assess the effect of MDM2 knockdown on the ability of HepG2 cells to form colonies. **D** Transwell assay was employed to verify the effect of MDM2 silencing on the migration of HepG2 cells. **E** RT-qPCR showing the expression of *SNAIL, SLUG*, *ZEB1*, and *ZEB2* following MDM2 knockdown, with *GAPDH* as an internal control. **F** RT-qPCR showing the transcript levels of *N-cadherin *and* Vimentin* in si-NTC and si-MDM2 transfected HepG2 cells, with GAPDH as an internal control. Data values are expressed as the mean ± SEM (*N* = 3) for all assays except for western blotting (*N* = 2). Statistically significant at *P < 0.05, ** *P* < 0.01, ***P < 0.001 (Student t-test, two-tailed). siMDM2-MDM2 siRNA, siNC- negative siRNA

To characterize the role of MDM2 in HepG2 cells, we explored the effect of MDM2 silencing on cell proliferation, migration, and EMT progression of HepG2 cells. Figure 4A&B illustrates that the on-target smart pool siRNAs induced remarkably strong and detectable knockdown (more than 80% at the mRNA level and almost 70% at the protein level) (Full length blot S3 A&B). The clonogenic cell survival and Transwell assays revealed that *MDM2* gene silencing impaired HepG2 cell proliferation and migration (Fig. 4C&D). The RT qPCR data indicated that MDM2 silencing significantly suppressed the transcript levels of EMT transcription factors such as SNAIL, SLUG, ZEB1, and ZEB2, and mesenchymal genes, such as N-cadherin and Vimentin (Fig. 4E&F).

## Discussion

Dysregulation of particular miRNAs has been reported in several types of cancer, including HCC [38]. The present study demonstrated that miR-590-3p is downregulated in HCC tissues and in different stages of HCC cell lines. Herein, we used HepG2 and SNU449 cell lines which are commonly used in HCC research, and their characteristics are well-described in the literature. HepG2 represent an early stage of HCC with an epithelial appearance, while SNU449 is considered an intermediate stage of HCC [29, 39]. We hypothesized that since miR-590-3p is downregulated in HCC compared to normal individuals, its expression is expected to decrease further as the tumor advances from an early stage to a later stage of the disease. Our findings revealed an almost 90-fold decrease in the expression level of miR-590-3p in SNU449 as compared to HepG2 cells. These results highlight the potential of miR-590-3p as a diagnostic and a prognostic factor in HCC, as it is able to differentiate between normal tissues and HCC, as well as between different stages of HCC.

However, it is important to consider the limitations of this study, such as the fact that miR-590-3p expression was only tested in HCC cell lines. Including normal liver cell lines would have provided a more comprehensive understanding of miR-590-3p expression in HCC tissue compared to normal tissue. Nonetheless, our study provides valuable insights into the function of miR-590-3p at different stages of HCC, which can guide future investigations into the potential clinical applications of this miRNA.

It is widely accepted that miRNAs play a significant role in cancer pathogenesis by regulating many biological processes such as proliferation, metastasis, and apoptosis [40]. In the Current study, the restoration of miR-590-3p by ectopic transfection of miRNA mimics reduces the clonogenicity and migration rates of the HepG2 cells. Moreover, overexpression of miR-590-3p caused a marked reduction in the transcript levels of EMT transcription factors (SNAIL, SLUG, ZEB1, and ZEB2*)* and the EMT mesenchymal marker, N-cadherin. Interestingly, the expression of Vimentin, another EMT mesenchymal marker, was not significantly affected by miR-590-3p overexpression. Collectively, these results suggest that miR-590-3p has tumor suppressive effects on HCC.

There is growing evidence indicating that miR-590-3p plays a role as a tumor suppressor or an oncomiR in several cancers. For example, miR-590-3p reduced migration, invasion and EMT in glioblastoma multiforme by targeting ZEB1 and ZEB2 [13], and in intrahepatic cholangiocarcinoma by targeting ZEB2 [41]. Similarly, the suppressive effects of miR-590-3p on apoptosis have been reported in the context of osteosarcoma [42] and breast cancer [43]. In contrast, miR-590-3p causes increased cellular proliferation and metastasis in some cancers, such as prostate [44], ovarian [14], gastric [45], and colorectal cancer [12]. However, the role of miR-590- 3p role in HCC remains unclear. Some studies have suggested that miR-590-3p targets the CHFR3/p-STAT3/p53 axis or activates the PI3K-AKT pathway, leading to a malignant phenotype [17, 18]. However, other reports have found that restoring miR-590-3p expression suppressed HCC growth via interaction with the *TEAD1* target gene or the *EED* gene [15, 16]. Two other studies suggested miR-590-3p played an intermediate tumor suppressor role in the LINC0016/miR-590-3p/ROCK and PART1/miR-590-3p/HMGB2 [19, 20]. These conflicting findings suggest a complex role for miR-590-3p in HCC pathogenesis, and further research is necessary to fully understand its mechanisms of action.

The discrepancies in the literature related to miR-590-3p likely arise from the fact that miRNAs can regulate multiple target genes, which can affect differential tumorigenic pathways. However, the concept that a single miRNA could function both as a tumor suppressor and an oncomiR in the same cancer is a relatively novel scientific idea.

The present study adds evidence to the tumor-suppressive role of miR-590-3p in HCC. It demonstrates the inhibition of three important cancer hallmarks: clonogenicity, migration, and EMT. Our findings support previous studies [15, 16, 19, 20] that suggest miR-590-3p has tumor suppressor effects in HCC. However, our results conflict with those of [17, 18], which suggest that miR-590-3p functions as an oncomiR in HCC. Thus, further research is required to reconcile these conflicting findings and fully understand the role of miR-590-3p in HCC.

A particularly interesting finding of the present study is the non-significant change in *Vimentin* gene expression in response to miR-590-3p overexpression. Because EMT is not a complete process in all cancers, a population of cancer cells will express mixed and different EMT markers [46]. Also, the desynchronization between EMT phenotype and marker gene expression has been reported before [47], with expression of some EMT markers remaining unchanged until after three days of treatment while the mesenchymal phenotype was detectable after just one day after treatment. Since *Vimentin* expression did not significantly change in our experiment, it is possible that Vimentin is not regulated by miR-590-3p as an EMT marker, or there may be intermediate players between miR-590-3p and Vimentin that slow down the signaling to a later stage of the EMT cascade. Further research is needed to clarify the role of miR-590-3p in regulating *Vimentin* expression in EMT.

The function of a particular miRNA in a certain cancer is mainly attributed to the function of its target genes [48]. Bioinformatic analysis revealed *MDM2* as a promising miR-590-3p candidate gene with a differential expression pattern between normal liver and HCC tissues. The upregulation of MDM2 in HCC tissues demonstrates a negative correlation with miR-590-3p downregulation, that was identified before, and was further corroborated by the reduction of mRNA *MDM2* levels after overexpressing miR-590-3p. The mechanistic interaction between MDM2 and miR-590-3p, established through the luciferase experiment, further confirms that MDM2 is a direct novel target of miR-590-3p in HCC. Collectively, these results suggest that there is a strong relationship between miR-590-3p and MDM2 that might be contributed, at least in part, to the suppressive effects of miR-590-3p on HCC discussed before.

We further examined the above hypothesis by knocking down MDM2 to gain insight into its biological role in HCC. *MDM2* is a major oncogene, which is expressed at high levels in several tumors including HCC [21, 22, 49, 50]. The main function of MDM2 is to influence the p53 tumor suppressor function through different mechanisms [51]. However, there is growing evidence suggesting that MDM2 interacts with certain proteins that contribute to cell proliferation, migration, EMT progression, and metastasis, independent of p53 [22, 24, 52, 53].

We observed that knocking down MDM2 suppressed HepG2 cell proliferation and migration, in line with these observations that have shown that MDM2 possesses proliferation- and migration-promoting activities in HCC and several other human cancers, independent of p53 [24, 25, 54]. Further evidence from studies on breast cancer suggests that MDM2 promotes cell motility and invasiveness of breast cancer cells by targeting E-cadherin for degradation and inducing MMP9 expression [55, 56]. Thus, the possibility that the MMPs and E-cadherin proteins are involved in the regulative effect of MDM2 on HepG2 cell migration cannot be excluded.

We demonstrated that MDM2 positively regulates *SNAIL* genes at the mRNA level, in contrast to previous studies that have shown MDM2 to negatively regulate SNAIL and SLUG at the protein level [57, 58]. Most previous studies focused on the protein targets of MDM2, and there is little information about the mRNA targets. Recent findings, however, demonstrated that MDM2 is also capable of binding to and stabilizing the mRNA of certain genes, including the EMT transcription factor, *SLUG* [59–62]. Notably, Jung et al. suggested that the expression of SLUG protein is determined by a balance between MDM2-mediated accumulation of *SLUG* mRNA and MDM2-mediated degradation of SLUG protein, with p53 required for the latter function [62]. In support of this assumption, our data implicate MDM2 serves as a positive regulator of the *SNAIL* family at the mRNA level in HepG2 cells. To the best of our knowledge, the current work is the first description of the regulative effect of MDM2 on *SNAIL* genes at the mRNA level in HCC.

The data presented here implies that MDM2 regulates the expression of ZEB factors in HCC. Consistent with this, MDM2 overexpression has been found to enhance the expression of EMT markers, such as ZEB1, via the B‐Raf signaling pathway in glioma, lung cancer, and breast cancer cells [63]. In addition, recent evidence has been published suggesting that MDM2 can regulate ZEB1 and ZEB2 by activating Smad2 and Smad3, resulting in EMT progression [50]. To the best of our knowledge, this is the first report of *ZEB2* regulation by MDM2 in the context of HCC. Our experimental results also suggested that silencing MDM2 mimics the tumor-suppressive activity of miR-590-3p in HepG2 cells, indicating that miR-590-3p plays a tumor-suppressor role in HepG2 by targeting MDM2.

It is important to note that siRNA-mediated knockdown of MDM2 can result in off-target effects, which can complicate the interpretation of results. This study utilized the On-TARGETplus SMARTpool siMDM2, a highly efficient and widely recognized approach to reduce off-target effects. Overall, our findings shed new light on the biological role of MDM2 in HCC and provided insights into potential therapeutic targets for this disease.

## Conclusion

**Fig. 5:**
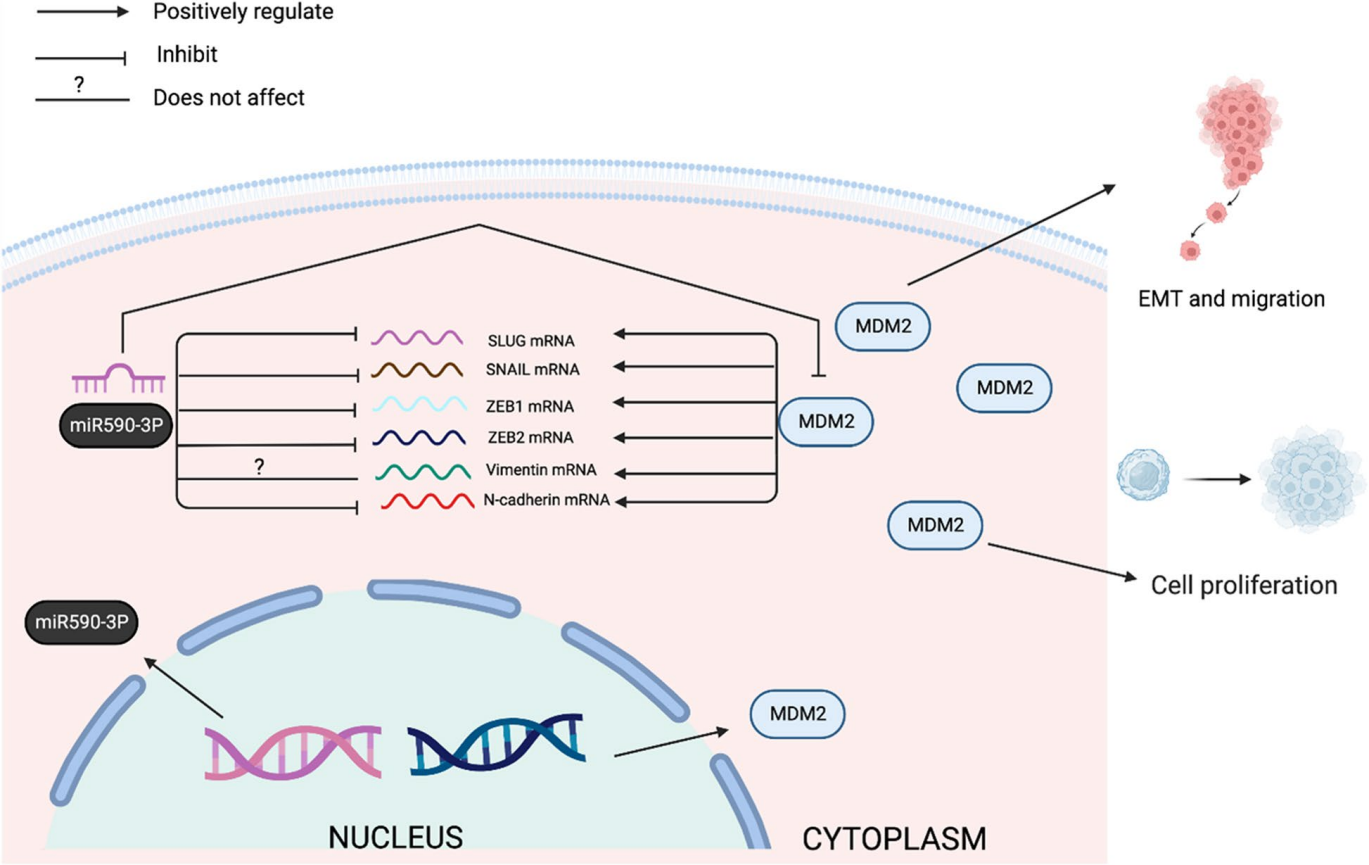
Hypothetical model for miR-590-3p/MDM2 axis role in HCC. *MDM2* acts as an oncogene in HCC. miR-590-3p exerts tumor suppressive roles in HCC by directly targeting MDM2*.* The figure was created with Biorender.com

This study presents a novel miR-590-3p/MDM2 axis (Fig. 5) through which the precise role of miR-590-3p in EMT regulation in HCC can be identified. Our findings suggest that the *MDM2* oncogene plays a significant role in HepG2 proliferation, migration, and EMT progression and that the tumor suppressive effect of miR-590-3p in HCC is due to interaction, at least in part, with MDM2. We believe that our findings could have important implications for HCC treatment considering the substantial role of the miR-590-3p/MDM2 axis in HCC pathogenesis, implicating it as an axis worthy of clinical investigation. Future studies on the correlation between miR-590-3p and MDM2 expression profiles with the clinico-pathological patterns in HCC might provide more insights into the significance of the miR-590-3p/ MDM2 axis as a potential therapeutic target of HCC. Finally, our results shed light on the importance of exploring more mRNA targets of MDM2 involved in HCC progression, which may help in understanding the metastasis of HCC through MDM2.

## Supplementary Information

**Additional file 1:** **Figure S1.** miR-590-3p directly targets EMT-TF SLUG, ZEB1, and ZEB2 in HCC, as determined by bioinformatics analysis (A) CSmiRTar database analysis showing *SLUG*, *ZEB1* and *ZEB2* as miR-590-3p potential target genes. (B) TargetScan analysis showing the sequence alignment between the seed region of miR-50-3p and its downstream target genes. Although *SNAIL* was not predicted as a direct target of miR-590-3p by CSmiRTar database or TargetScan, we observed a marked reduction in the transcript levels of *SNAIL *using RT-qPCR analysis, suggesting that miR-590-3p may target *SNAIL *through other players primarily regulated by miR-590-3p, such as MDM2. * SNAI2= SLUG.

**Additional file 2:** **Figure S2.** Sequence alignment between miR-590-3p and N-cadherin but not Vimentin. TargetScan shows that the mesenchymal marker N-cadherin harbours three different binding sites with miR-590-3p in its 3′UTR, while no binding was shown with the other tested mesenchymal marker, Vimentin, supporting the notion that miR-590-3p suppresses EMT through directly regulating N-cadherin, while it might be that there is an indirect regulation on Vimentin hindering the early detection of the change in its levels, that is why it was not detected in our analysis.

**Additional file 3:** **Figure (S3).** MDM2 siRNA effectively silences MDM2 expression at the protein levels. Western blot uncropped full-length blot for A) first biological replicate and B) second biological replicate.

## Data Availability

All data generated or analyzed during this study are included in this published article.

## Abbreviations

DMEM: Dulbecco's Modified Eagle's Medium
FBS: Fetal bovine serum
PBS: Phosphate buffered saline
SDS: Sodium Dodecyl Sulfate
TBST: 0.01% Tween-20 in 1X TBS
miRNA590-3p: miRNA590-3p
siRNA: small interfering RNA
SNAIL: Snail family zinc finger 1
N-cadherin: Neural Cadherin
MDM2: Murine double minute 2
ZEB: Zinc finger E-box-binding homeobox
EMT: Epithelial‐mesenchymal transition
HCC: Hepatocellular carcinoma
RT‐qPCR: Reverse transcription‐quantitative PCR
siMDM2: MDM2 siRNA
siNTC: negative siRNA
