# Supplementary material for: Functional role and epithelial to mesenchymal transition of the miR-590-3p/MDM2 axis in hepatocellular carcinoma
Source: BMC Cancer. 2023 May 4;23:396. doi: 10.1186/s12885-023-10861-y (PMC10157954; doi:10.1186/s12885-023-10861-y)
Supplement: Supplementary file 1 — Additional file 1: Figure S1. miR-590-3p directly targets EMT-TF SLUG, ZEB1, and ZEB2 in HCC, as determined by bioinformatics analysis (A) CSmiRTar database analysis showing SLUG, ZEB1 and ZEB2 as miR-590-3p potential target genes. (B) TargetScan analysis showing the sequence alignment between the seed region of miR-50-3p and its downstream target genes. Although SNAIL was not predicted as a direct target of miR-590-3p by CSmiRTar database or TargetScan, we observed a marked reduction in the transcript levels of SNAIL using RT-qPCR analysis, suggesting that miR-590-3p may target SNAIL through other players primarily regulated by miR-590-3p, such as MDM2. * SNAI2= SLUG. [file 12885_2023_10861_MOESM1_ESM.docx]

**
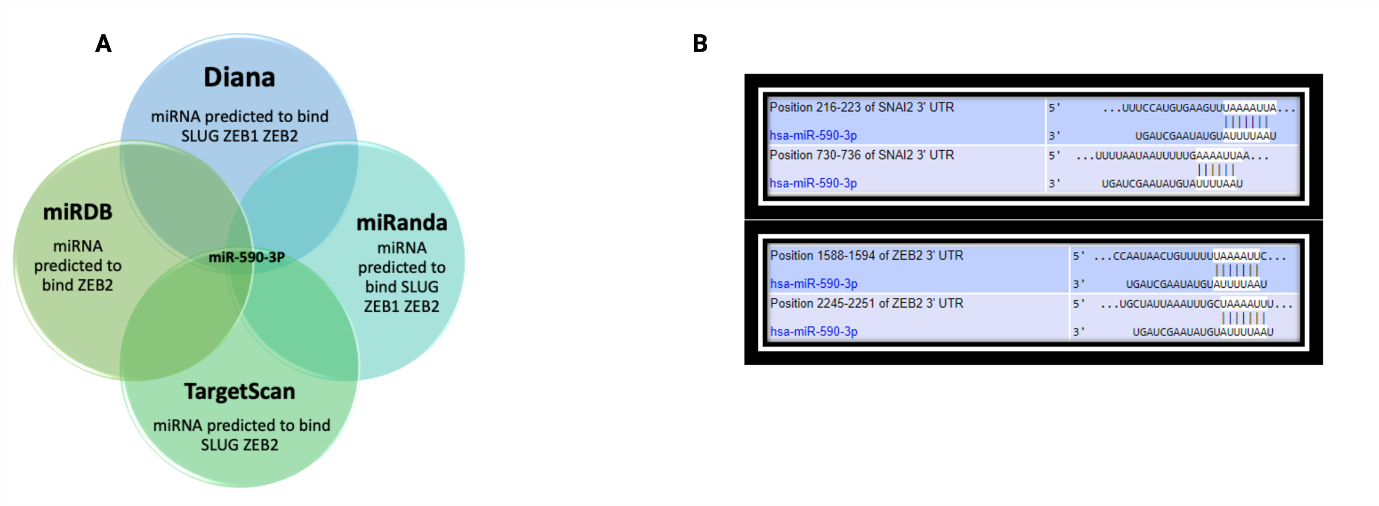
Aditional file 1**

**Figure S1: miR-590-3p directly targets EMT-TF SLUG, ZEB1, and ZEB2 in HCC, as determined by bioinformatics analysis** (A) CSmiRTar database analysis showing *SLUG*, *ZEB1* and *ZEB2* as miR-590-3p potential target genes. (B) TargetScan analysis showing the sequence alignment between the seed region of miR-50-3p and its downstream target genes. Although *SNAIL* was not predicted as a direct target of miR-590-3p by CSmiRTar database or TargetScan, we observed a marked reduction in the transcript levels of *SNAIL* using RT-qPCR analysis, suggesting that miR-590-3p may target *SNAIL* through other players primarily regulated by miR-590-3p, such as MDM2. * SNAI2= SLUG
