# Supplementary material for: Functional role and epithelial to mesenchymal transition of the miR-590-3p/MDM2 axis in hepatocellular carcinoma
Source: BMC Cancer. 2023 May 4;23:396. doi: 10.1186/s12885-023-10861-y (PMC10157954; doi:10.1186/s12885-023-10861-y)
Supplement: Supplementary file 2 — Additional file 2: Figure S2. Sequence alignment between miR-590-3p and N-cadherin but not Vimentin. TargetScan shows that the mesenchymal marker N-cadherin harbours three different binding sites with miR-590-3p in its 3′UTR, while no binding was shown with the other tested mesenchymal marker, Vimentin, supporting the notion that miR-590-3p suppresses EMT through directly regulating N-cadherin, while it might be that there is an indirect regulation on Vimentin hindering the early detection of the change in its levels, that is why it was not detected in our analysis. [file 12885_2023_10861_MOESM2_ESM.docx]

## Additional File 2

##
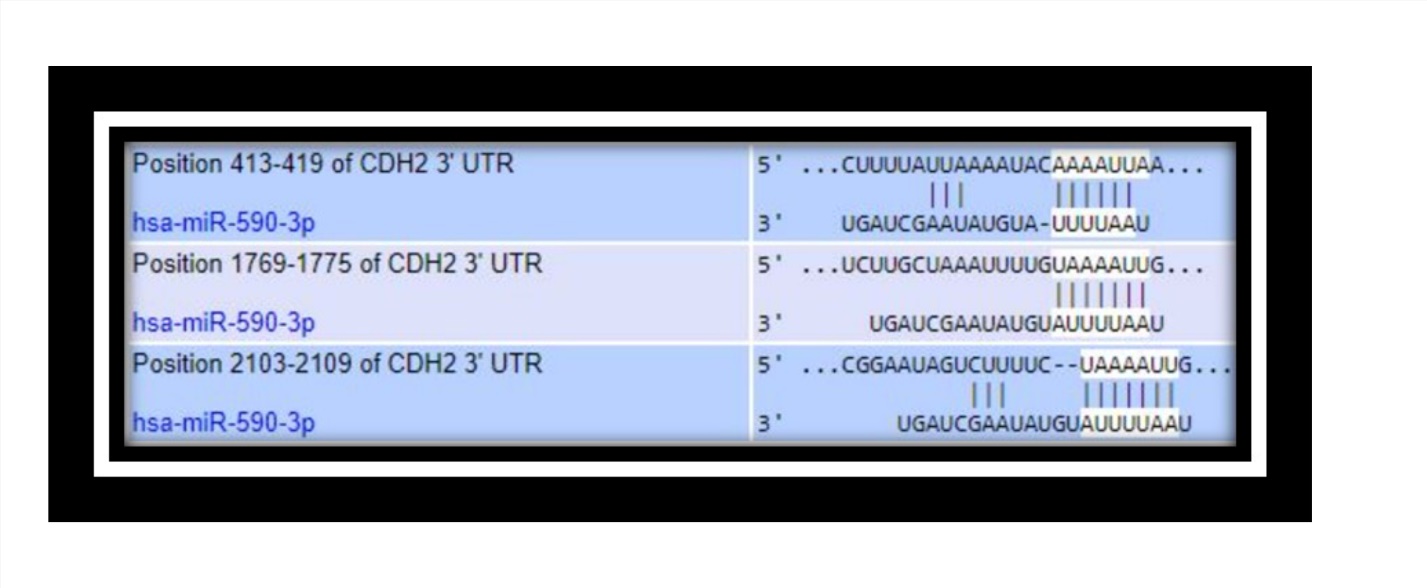


**Figure S2: Sequence alignment between miR-590-3p and N-cadherin but not Vimentin.** TargetScan shows that the mesenchymal marker N-cadherin harbours three different binding sites with miR-590-3p in its 3′UTR, while no binding was shown with the other tested mesenchymal marker, Vimentin, supporting the notion that miR-590-3p suppresses EMT through directly regulating N-cadherin, while it might be that there is an indirect regulation on Vimentin hindering the early detection of the change in its levels, that is why it was not detected in our analysis
