# Supplementary material for: Functional role and epithelial to mesenchymal transition of the miR-590-3p/MDM2 axis in hepatocellular carcinoma
Source: BMC Cancer. 2023 May 4;23:396. doi: 10.1186/s12885-023-10861-y (PMC10157954; doi:10.1186/s12885-023-10861-y)
Supplement: Supplementary file 3 — Additional file 3: Figure (S3). MDM2 siRNA effectively silences MDM2 expression at the protein levels. Western blot uncropped full-length blot for A) first biological replicate and B) second biological replicate. [file 12885_2023_10861_MOESM3_ESM.docx]

**Additional file 3**

**Western blot uncropped full-length blots**

**A**


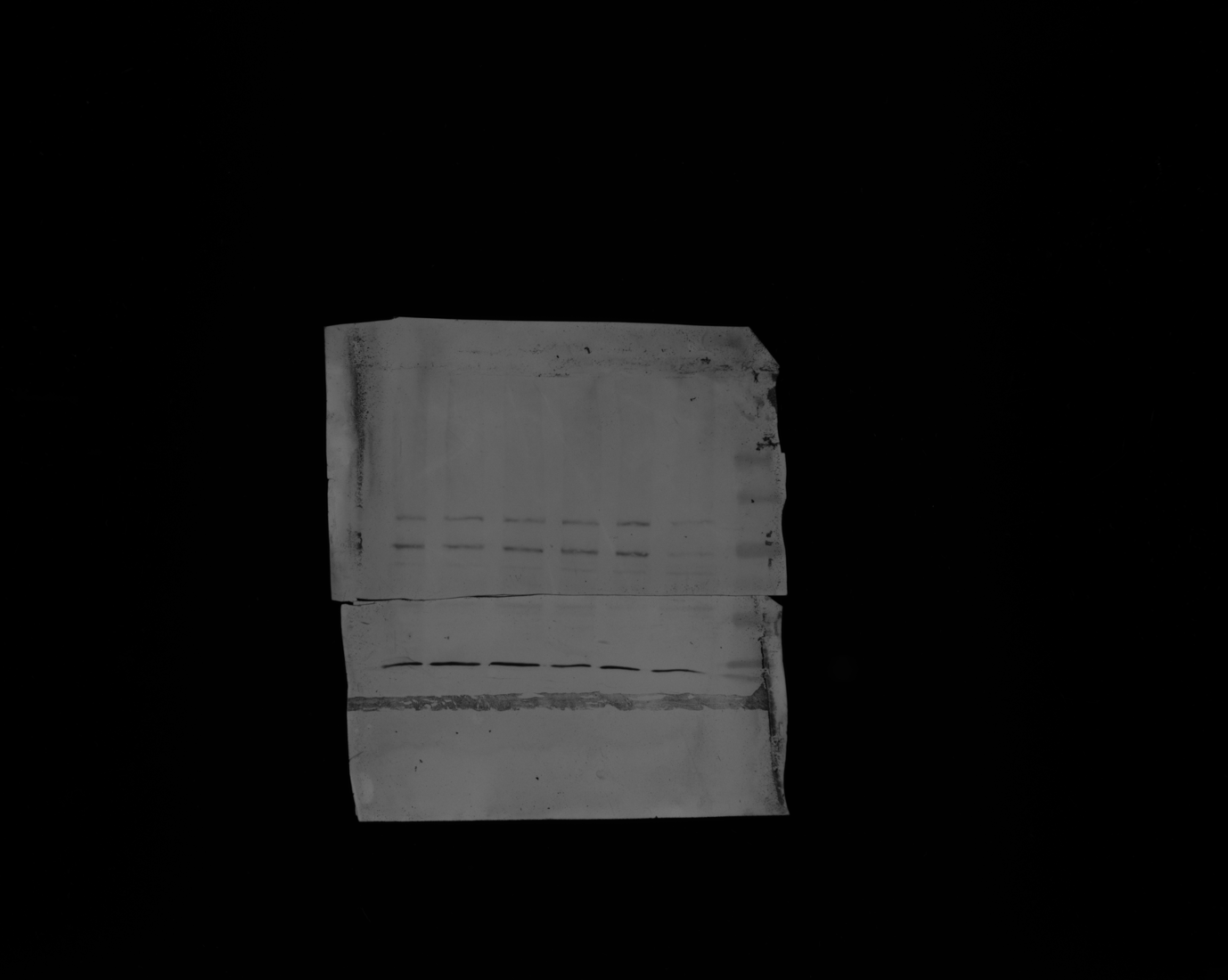


GAPDH

MDM2

Si-NTC

si-MDM2

Laddar

siMDM2-rep2

siNTC

**B**

Laddar

siMDM2- rep1


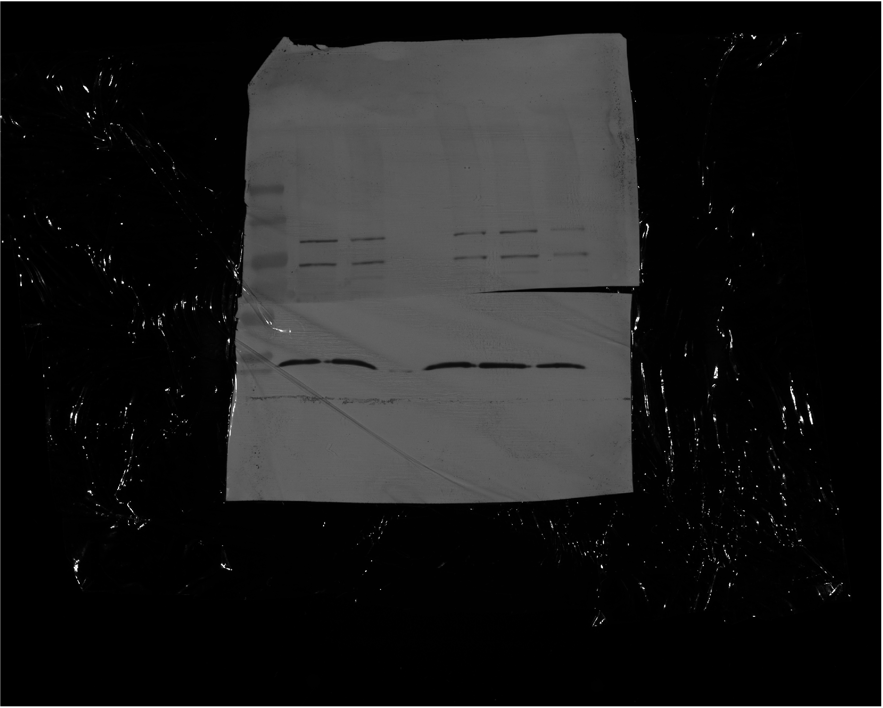


GAPDH

MDM2

Figure (S3) MDM2 siRNA effectively silences MDM2 expression at the protein levels.

Western blot uncropped full-length blot for A) first biological replicate and B) second biological replicate.
